# Amelioration of signaling deficits underlying metabolic shortfall in TREM2^R47H^ human iPSC‐derived microglia

**DOI:** 10.1111/febs.17353

**Published:** 2024-12-26

**Authors:** Foteini Vasilopoulou, Thomas M. Piers, Jingzhang Wei, John Hardy, Jennifer M. Pocock

**Affiliations:** ^1^ Department of Neuroinflammation UCL Queen Square Institute of Neurology, University College London UK; ^2^ Department of Neurodegenerative Disease UCL Queen Square Institute of Neurology London UK; ^3^ UK Dementia Research Institute UCL Queen Square Institute of Neurology London UK; ^4^ Reta Lila Weston Institute UCL Queen Square Institute of Neurology London UK; ^5^ NIHR University College London Hospitals Biomedical Research Centre and Institute for Advanced Study The Hong Kong University of Science and Technology China; ^6^ Present address: RD&E Hospital Wonford University of Exeter Medical School Exeter UK; ^7^ Present address: The Institute of Anatomy University Medical Center Mainz & Leibniz Institute for Resilience Research (LIR) Mainz Germany

**Keywords:** Alzheimer's disease, human microglia, metabolism, neurodegeneration, R47H TREM2 variant, TCA‐metabolites

## Abstract

The microglial triggering receptor expressed on myeloid cells 2 (TREM2) is required for diverse microglia responses in neurodegeneration, including immunometabolic plasticity, phagocytosis, and survival. We previously identified that patient iPSC‐derived microglia (iPS‐Mg) harboring the Alzheimer's disease (AD) TREM2^R47H^ hypomorph display several functional deficits linked to metabolism. To investigate whether these deficits are associated with disruptions in metabolite signaling, we generated common variant, TREM2^R47H^ and TREM2^−/−^ variant human iPS‐Mg. We assessed the ability of supplementation with citrate or succinate, key metabolites and cell cycle breaking points upon microglia activation, to overcome these functional deficits with potential impact on neurons. Succinate supplementation was more effective than citrate at overcoming mitochondrial deficits in OXPHOS and did not promote a glycolytic switch. Citrate enhanced the lipid content of TREM2^R47H^ iPS‐Mg and was more effective at overcoming Αβ phagocytic deficits, whereas succinate increased lipid content and phagocytic capacity in TREM2^−/−^ iPS‐Mg. Microglia cytokine secretion upon pro‐inflammatory activation was moderately affected by citrate or succinate showing a condition‐dependent increasing trend. Neither metabolite altered basal levels of soluble TREM2 shedding. In addition, neither citrate nor succinate enhanced glycolysis; instead, drove their effects through oxidative phosphorylation. IPS‐neurons exposed to conditioned medium from TREM2 variant iPS‐Mg showed changes in oxidative phosphorylation, which could be ameliorated when iPS‐Mg were first treated with citrate or succinate. Our data point to discrete pathway linkage between microglial metabolism and functional outcomes with implications for AD pathogenesis and treatments.

## Graphical abstract

The microglial receptor TREM2 is required for microglia responses in neurodegeneration, and the loss‐of‐function mutation R47H results in functional deficits linked to metabolism. Using human TREM2^R47H^ or TREM2^−/−^ microglia we show that supplementation with either citrate or succinate ameliorates microglia deficiencies due to TREM2 deficiency, including oxidative phosphorylation (OXPHOS), phagocytosis or lipid metabolism, and restores neuronal OXPHOS via secretome products. These findings provide insights into the metabolic pathways that underlie TREM2‐related metabolic disturbances in microglia.

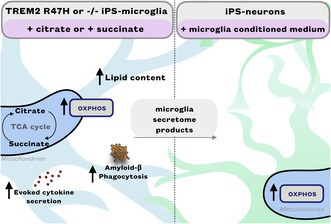

**Abbreviations**
2‐DG: 2‐deoxyglucose
AD: Alzheimer's disease
APOE: apolipoprotein E
ATP: adenosine triphosphate
CV: common variant
DAM: disease‐associated microglia
HBSS: Hepes‐buffered saline solution
HIF1α: hypoxia inducible factor 1 α
iPS‐Mg: induced pluripotent stem cell‐derived microglia
LoF: loss‐of‐function
MCM: microglia conditioned medium
NMM: neuronal maintenance media
OCR: oxygen consumption rates
OXPHOS: oxidative phosphorylation
PER: proton efflux rate
SOD2: superoxide dismutase 2
sTREM2: soluble TREM2
TCA: tricarboxylic acid
TREM2: triggering receptor expressed on myeloid cells

## Introduction

Variants in the triggering receptor expressed on myeloid cells (TREM2) are linked to an increased risk of developing dementia, including late‐onset Alzheimer's disease (AD) [1, 2]. TREM2 is expressed i microglia, the resident immune cells of the brain, and is required for diverse microglia responses in neurodegeneration, including immunometabolic plasticity, phagocytosis, and survival [3, 4, 5, 6]. Identifying how TREM2 variants affect the function of microglia is pertinent to our understanding of disease progression in AD and for identifying new pathways for protection.

In a surveillant or homeostatic state, microglia express a reduced protein and pathway activation load. Upon input of appropriate signals, microglia rapidly respond with increased energy production, gene expression, and protein synthesis [7]. In this regard, microglia switch from a dependency on oxidative phosphorylation (OXPHOS) to glycolysis, which, whilst producing less energy (adenosine triphosphate, ATP) compared with OXPHOS, has an ATP production rate that is faster upon insult [8, 9, 10]. In TREM2^−/−^ AD animal models, a reduced metabolic ‘fitness’ is associated with TREM2 loss of function [6]. Our previous studies in human induced pluripotent stem cell‐derived microglia (iPS‐Mg) from patients expressing the AD‐linked R47H^het^ TREM2 variant and the Nasu Hakola variants T66M^hom^ and W50C^hom^ increased our understanding of the deficits linked to TREM2 loss‐of‐function (LoF) variants; specifically in aerobic glycolysis and mitochondrial respiration [11, 12, 13]. Moreover, we reported that iPS‐Mg expressing AD‐linked TREM2 variants are unable to switch from a mainly OXPHOS state of energy production to one in which glycolysis dominates [12]. We also identified that this inability to generate sufficient energy results in changes to the exosome profile of these cells [14, 15] and an inability to activate the inflammasome [13].

Recent findings indicate that signaling metabolites play a pivotal role in modulating microglial functions through diverse mechanisms [16, 17, 18, 19]. At the same time, the activation of immune cells has been associated with tricarboxylic acid (TCA) cycle fragmentation downstream of two key signaling metabolites, namely citrate and succinate [20, 21]. While citrate can bridge carbohydrate and fatty acid metabolism in the TCA cycle, succinate interacts directly with the mitochondrial electron transport chain, enabling a ‘shortcut’ route to ATP production via oxidative metabolism [22, 23]. While TREM2 impact on energetic and anabolic pathways has been linked to alterations in glycolytic and TCA cycle intermediates, including citrate and succinate, which are reduced in TREM2^−/−^ macrophages [6] the involvement of those energy substrates in TREM2‐dependent metabolic reprogramming pathways is still underexplored.

Here we hypothesized that microglia metabolic deficits in TREM2 LoF variants may be linked to metabolite signaling deficits, and in turn, fuelling microglia with these metabolites could restore the functional deficiencies exhibited by TREM2 LoF variant microglia with potential impact on neurons. To address that, we determined the impact of supplementing culture media with citrate or succinate on functions found to be deficient in TREM2 LoF variant human iPS‐Mg. Considering that the metabolic profile is closely related to the status and functional changes of microglia, this may allow a remedial approach to TREM2‐linked deficits. We show that citrate and succinate supplementations affect microglia metabolic processes and restore key functions, providing insights into the metabolic pathways that underly TREM2‐related metabolic disturbances in microglia.

## Results

### TREM2 variant deficits in maximal respiration and spare respiratory capacity are rescued with succinate but not citrate

**Fig. 1:**
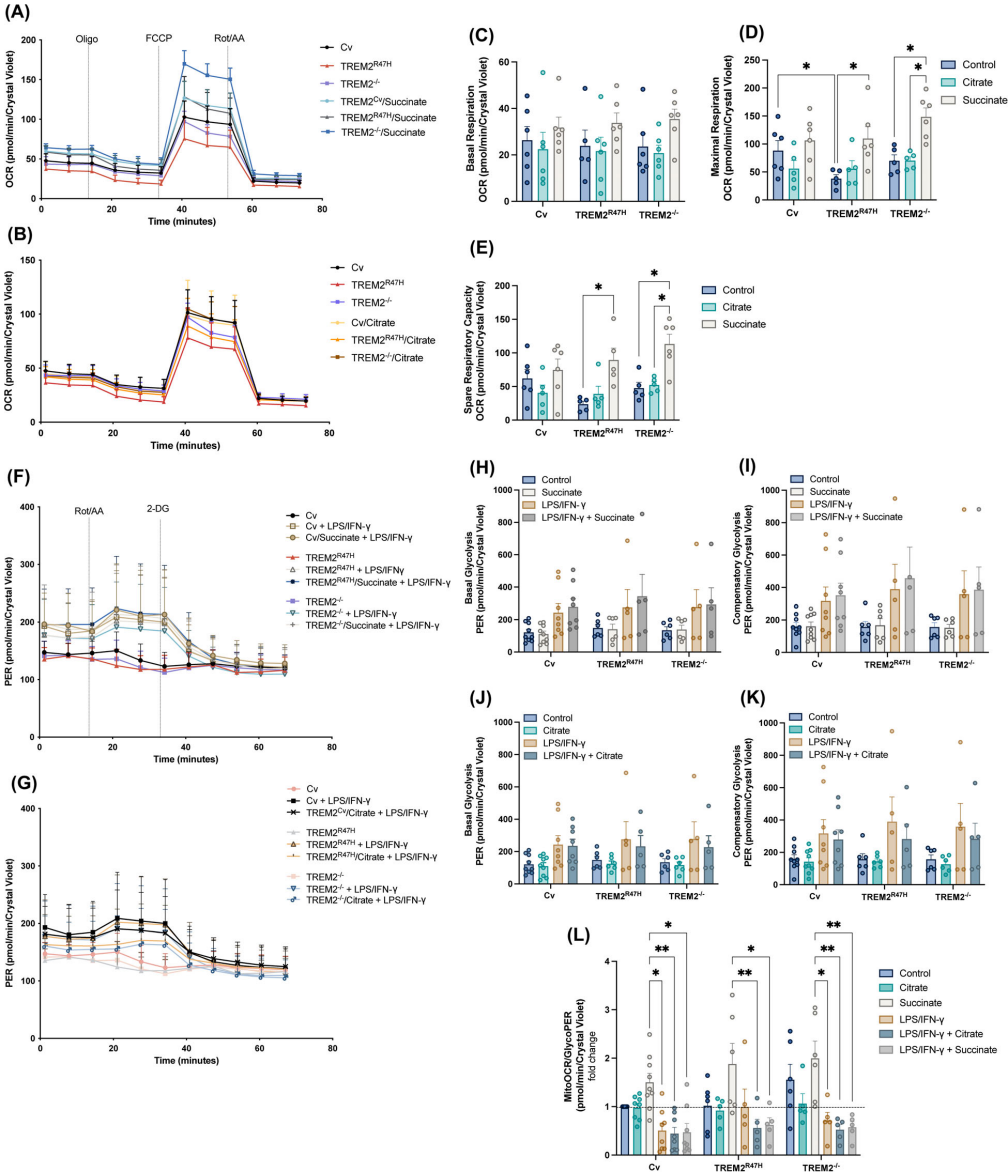
Metabolic function of iPS‐Mg supplemented with citrate or succinate. (A, B) Metabolic analysis of oxygen consumption rate (OCR) by Searhorse, that allows evaluation of oxidative phosphorylation in live cells upon injections with Oligomycin (Oligo), FCCP, Rotenone (Rot) and Antimycin (AA) (C) basal respiration, (D) maximal respiration, (E) spare respiratory capacity in Common variant (Cv), TREM2^R47H^ and TREM2^−/−^ iPS‐Mg after 24 h treatment with succinate (10 mm) or citrate (10 mm) (*n* = 5–6 biological replicates; one‐way ANOVA to compare controls or two‐way ANOVA to compare controls with treated groups, following Tukey *post‐hoc* analysis). (F, G) Metabolic analysis of proton efflux by Seahorse, that allows measurements of the glyocolytic rates for basal conditions and compensatory glyocolysis following mitochondrial inhibition, upon injections with Rot/AA and 2‐Deoxyglucose (2‐DG) (H, J) basal glycolysis, (I, K) compensatory glycolysis, and (L) mitoOCR/GlycoPER ratio in basal conditions and upon LPS/IFN‐γ stimulation in iPS‐Mg expressing Cv, TREM2^R47H^ or TREM2^−/−^ after incubations with citrate (10 mm) or succinate (10 mm) (*n* = 5–9 biological replicates; one‐way ANOVA to compare controls or two‐way ANOVA to compare controls with treated groups, following Tukey *post‐hoc* analysis). Data are presented as mean ± SEM. *P < 0.05, **P < 0.01.

We performed Seahorse metabolic analysis of oxidative phosphorylation (OCR) in iPS‐Mg expressing TREM2 Cv, TREM2^R47H^, or TREM2^−/−^ after a 24 h preincubation with media supplemented with succinate (10 mm) (Fig. 1A) or citrate (10 mm) (Fig. 1B). The results indicated that deficits in maximal respiration and spare respiratory capacity exhibited in iPS‐Mg expressing the TREM2^R47H^ were rescued when cells were pre‐incubated with succinate but not with citrate (Fig. 1C–E). In TREM2^−/−^ iPS‐Mg, only succinate supplementation further increased these parameters (Fig. 1C–E). None of the substrates significantly affected the basal respiration in Cv iPS‐Mg (Fig. 1C). LPS/IFN‐γ stimulation for 24 h increased the OCR parameters evaluated in all iPS‐Mg lines tested (Fig. S2A–C).

### Compensatory glycolysis is not affected by citrate or succinate

Metabolic analyses of the glycolytic rate (PER) in iPS‐Mg with Cv, TREM2^R47H^, or TREM2^−/−^ (Fig. 1F,G) showed that after 24 h preincubation, LPS/IFN‐γ promoted a swift increase in glycolysis as shown by a decrease in MitoOCR/GlycoPER ratio in all iPS‐Mg genotypes (Fig. 1L). Succinate or citrate treatments, however, did not affect basal glycolysis (Fig. 1H,J) or compensatory glycolysis (Fig. 1I,K).

### Lipid deficits are differentially modified upon treatment with citrate and succinate

**Fig. 2:**
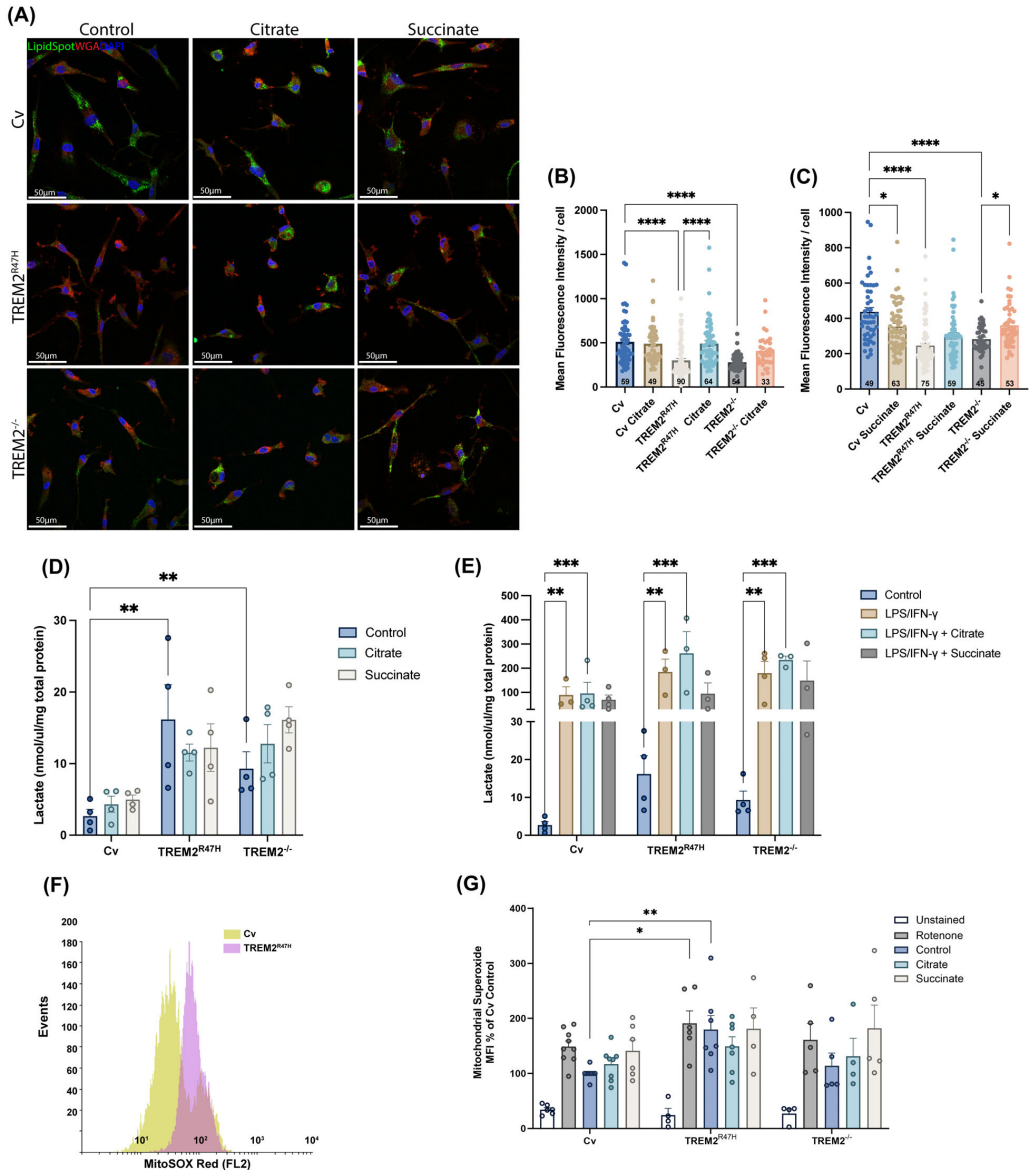
Lipid content, lactate and mitochondrial superoxide production in iPS‐Mg supplemented with citrate or succinate. (A) Lipid content was evaluated by LipidSpot™488 staining of lipid droplets by confocal imaging, and (B, C) mean fluorescence intensity of the lipid droplets was quantified in Common variant (Cv), TREM2^R47H^ and TREM2^−/−^ variant iPS‐Mg after medium supplementation with citrate (10 mm) or succinate (10 mm) for 24 h (*n* = 3 biological replicates, the number of cells analyzed per group is indicated at the bottom of the bar chart). Scale bars = 50 μm. (D) Basal lactate production and (E) lactate production upon LPS/IFN‐γ stimulation were determined in the iPS‐Mg cell culture medium after supplementation with citrate or succinate (*n* = 3–4 biological replicates). (F) Representative histogram and (G) mitochondrial superoxide (MitoSOX) production as determined by FACS in Cv, TREM2^R47H^ and TREM2^−/−^ variant iPS‐Mg after succinate or citrate supplementation or rotenone treatment (positive control) (*n* = 5–9 biological replicates). Data are presented as mean ± SEM. Statistical significance for (A), (B), (D), (E), (G) was addressed using one‐way ANOVA to compare controls or two‐way ANOVA to compare controls with treated groups, following Tukey *post‐hoc* analysis. *P < 0.05, **P < 0.01, ***P < 0.001; ****P < 0.0001.

TREM2 is a modulator of lipid metabolism [24]. To determine the influence of citrate or succinate on the lipid content of iPS‐Mg with TREM2 variants, we analyzed lipid droplet content in these cells by Lipid Spot staining (Fig. 2A). TREM2^R47H^ or TREM2^−/−^ iPS‐Mg showed a significant reduction in overall lipid droplet content/cell compared with Cv iPS‐Mg (Fig. 2B,C). Treatment with citrate (10 mm) did not significantly affect the lipid content/cell of Cv or TREM2^−/−^ iPS‐Mg (Fig. 2B) but significantly enhanced the lipid content in TREM2^R47H^ iPS‐Mg. Succinate produced a significant but small drop in lipid drop content in Cv, did not significantly affect it in TREM2^R47H^, but produced a slight but significant increase in TREM2^−/−^ iPS‐Mg (Fig. 2C).

### Lactate metabolism is enhanced with LPS/IFN‐γ treatment but not modified with citrate or succinate

As lactate is a waste product of cells with a high metabolic rate and is implicated in immunometabolism of microglia [25] as well as neurodegeneration [26], the secretion of both lactate enantiomers from iPS‐Mg was investigated, since both d‐lactate and l‐lactate are produced by microglia [26]. Interestingly the levels of l‐lactate secreted were different across the TREM2 variants at basal levels, with more lactate secreted by TREM^R47H^ and TREM2^−/−^ iPS‐Mg compared with Cv iPS‐Mg (Fig. 2D), and significantly enhanced in all variants when the cells were incubated with LPS/IFN‐γ for 24 h (Fig. 2E). However, neither citrate nor succinate significantly altered l‐lactate secretion across any of the iPS‐Mg lines (Fig. 2D,E). d‐lactate was not secreted at basal conditions to detectable levels (data not shown).

### Mitochondrial superoxide production is not modulated by citrate or succinate

We showed previously that iPS‐Mg expressing TREM2^R47H^ heterozygous produced significantly more mitochondrial‐generated superoxide than TREM2 Cv expressing microglia at basal levels [12]. This was also the case here for TREM2^R47H^ homozygous compared with Cv (Fig. 2F,G) whereas superoxide production increase in TREM2^−/−^ iPS‐Mg was slight but not significant (Fig. 2G). Media supplementation with citrate or succinate for 24 h did not significantly modify superoxide production across any of the lines (Fig. 2G).

### Deficits in Aβ_1–42_ uptake are restored by citrate

**Fig. 3:**
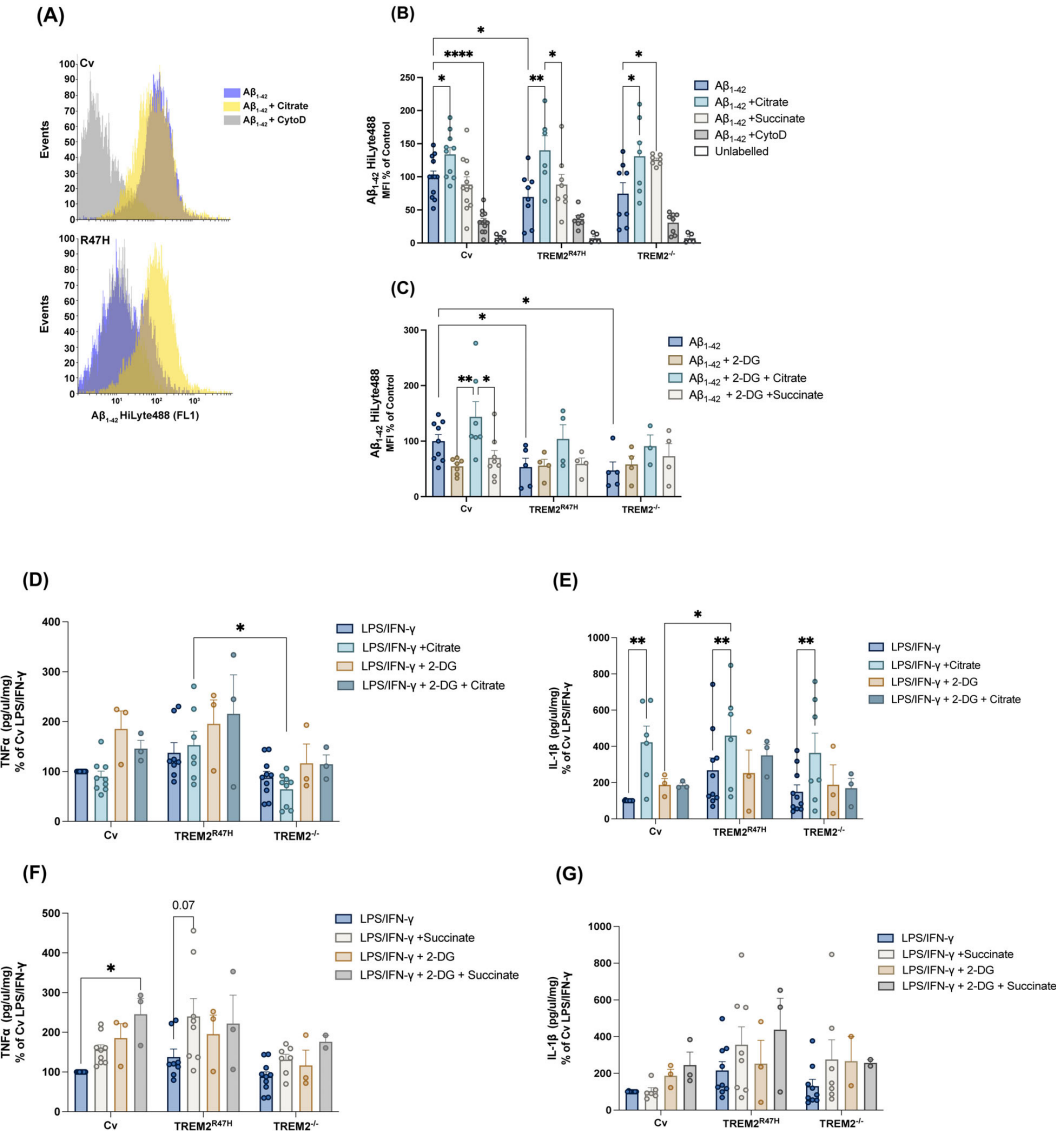
Effect of citrate and succinate on Aβ_1–42_ phagocytosis and cytokine secretion by iPS‐Mg. (A) Representative histograms of Aβ_1–42_ uptake in iPS‐Mg upon citrate supplementation for 24 h. (B) The ability of common variant (Cv), TREM2^R47H^ and/or TREM2^−/−^ variant iPS‐Mg to phagocytose Aβ_1–42_ oligomers was assessed after citrate and succinate supplementation in basal conditions (*n* = 7–12 biological replicates) and (C) upon inhibition of glycolysis by 2‐Deoxyglucose (2‐DG) (*n* = 3–9 biological replicates). CytoD, an inhibitor of actin‐mediated phagocytosis, was used as a negative control for Aβ_1–42_ uptake. (D, E) LPS/IFN‐γ evoked pro‐inflammatory release of TNFα (*n* = 3–10 biological replicates) and (F, G) IL‐1β were assessed by ELISA in the culture medium of iPS‐Mg treated with citrate or succinate in basal conditions and after glycolytic inhibition by 2‐DG (*n* = 2–10 biological replicates). Data are presented as mean ± SEM. Statistical significance was addressed for (B), (C), (D), (F), (G) using one‐way ANOVA to compare LPS/IFN‐γ and Aβ_1–42_ control groups or two‐way ANOVA to compare controls LPS/IFN‐γ and Aβ_1–42_ controls with treated groups, with Tukey *post‐hoc* analysis; *P < 0.05, **P < 0.01, ****P < 0.0001.

Previously we and others have identified an aberrant phagocytic phenotype in iPS‐Mg expressing TREM2 LoF variants, both in their ability to phagocytose apoptotic cells [11] and in their ability to phagocytose Aβ_1–42_ [12]. Here preincubation with citrate but not succinate was able to restore the impaired Aβ_1–42_ phagocytosis in TREM2^R47H^ iPS‐Mg (Fig. 3A,B), and both metabolites increased Aβ_1–42_ uptake in TREM2^−/−^ iPS‐Mg (Fig. 3B). Inhibition of glycolysis by 2‐DG led to impaired Aβ_1–42_ phagocytosis in Cv iPS‐Mg which was restored by citrate but not by succinate (Fig. 2C). Furthermore, citrate was unable to reverse the effects of glycolytic inhibition by 2‐DG in TREM2^R47H^ and TREM2^−/−^ iPS‐Mg and succinate had no effect (Fig. 2C).

### Inflammatory cytokine secretion is differentially modified by citrate and succinate

Since the secretion of inflammatory cytokines is implicated in AD [27, 28] and at the same time, metabolic intermediates can act as pro‐inflammatory agents *per se* [16, 23, 29, 30], we investigated whether TNFα or IL‐1β secretion is modulated by medium supplementation with succinate or citrate (Fig. 3D–G). Basal levels of TNFα or IL‐1β were very low (Fig. S2D,E) and were not altered by citrate or succinate (data not shown), thus we investigated the modulation of evoked cytokine secretion. Citrate did not significantly modify TNFα secretion evoked with LPS/IFN‐γ in any of the lines or following co‐treatment with 2‐DG compared with untreated cells (Fig. 3D). However, TNFα secretion was significantly lower in TREM2^−/−^ compared with TREM2^R47H^ upon citrate supplementation (Fig. 3D). Furthermore, citrate significantly enhanced LPS/IFN‐γ evoked IL‐1β secretion, but not that of 2‐DG (Fig. 3E). Succinate increased LPS/IFN‐γ evoked TNFα secretion in Cv iPS‐Mg which was significantly higher upon 2‐DG inhibition (Fig. 3F). Similarly, succinate elevated secretion in TREM2^R47H^ iPS‐Mg without blocking glycolysis with 2‐DG (Fig. 3F). TREM2^−/−^ lines showed no significant change in TNFα secretion with succinate supplementation in any condition (Fig. 3F). There was no significant effect of succinate on IL‐1β secretion in any treatment or TREM2 line compared with LPS/IFN‐γ alone (Fig. 3G).

### Soluble TREM2 shedding is not reduced by citrate or succinate

**Fig. 4:**
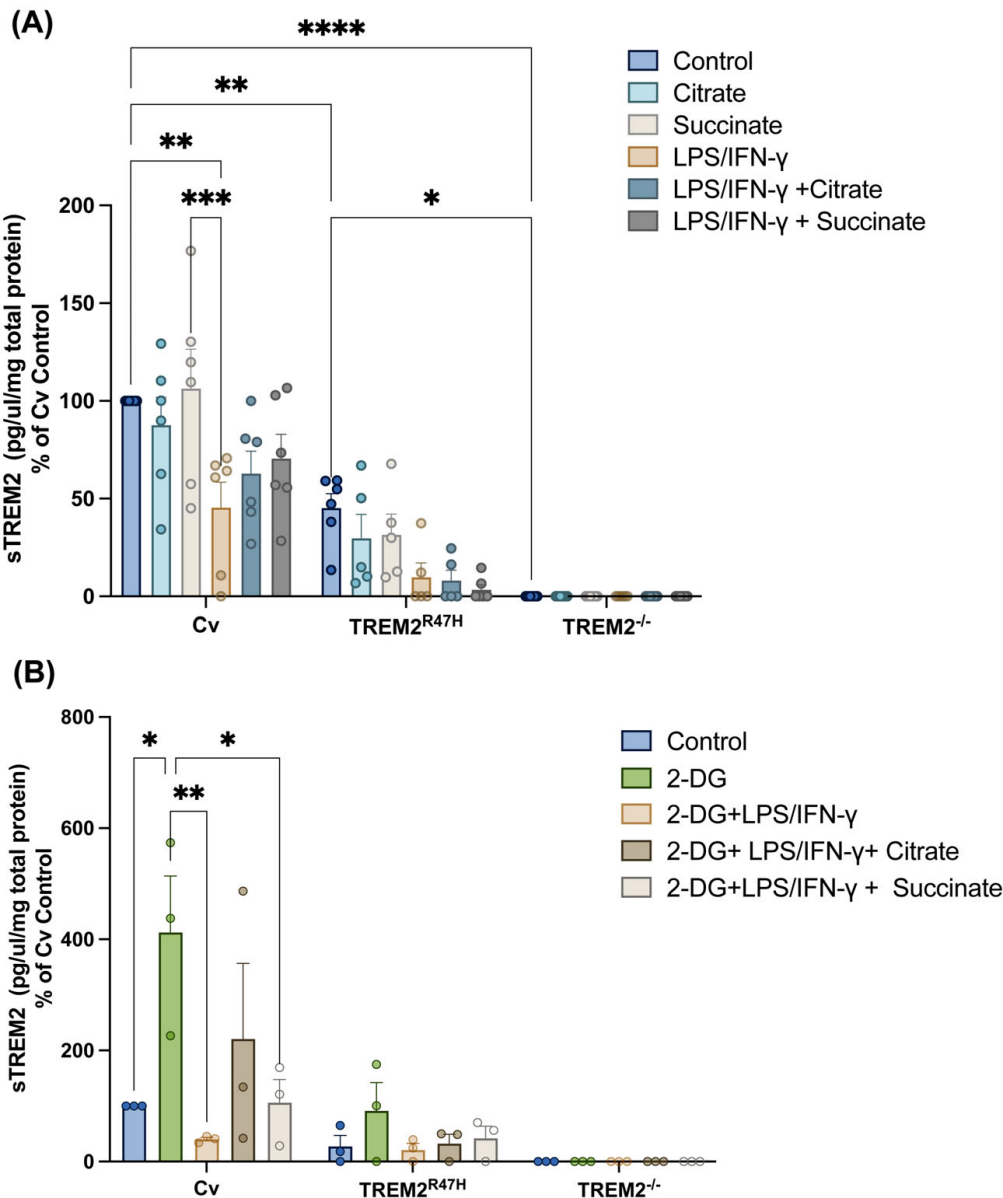
Effect of citrate or succinate on shed TREM2 in iPS‐Mg in basal conditions and upon microglia stimulation. (A) Shed soluble TREM2 (sTREM2) was determined in the culture medium of unstimulated common variant (Cv), TREM2^R47H^ and TREM2^−/−^ iPS‐Mg after treatments with citrate or succinate (*n* = 5–6 biological replicates) and/or (B) 2‐DG mediated inhibition of glycolysis in basal conditions and upon LPS/IFNγ stimulation (*n* = 3 biological replicates). Data are presented as mean ± SEM. Statistical significance was addressed using two‐way ANOVA with Tukey *post‐hoc* analysis; *P < 0.05, **P < 0.01, ***P < 0.001; ****P < 0.0001.

Since sTREM2 shedding is reduced in TREM2^R47H^ variants, we investigated the effects of citrate or succinate on this iPS‐Mg variant as well as on Cv. TREM2^−/−^ iPS‐Mg served as negative controls for sTREM2 shedding. As expected, sTREM2 secretion was significantly reduced in Cv and TREM^R47H^ following LPS/IFN‐γ stimulation compared with basal levels (Fig. 4A). Furthermore, less sTREM2 was shed at basal or following stimulation in TREM2^R47H^ compared with TREM2^Cv^ iPS‐Mg (Fig. 4A). Citrate did not significantly modify the shedding of sTREM2 in either Cv or R47H expressing iPS‐Mg at basal or evoked conditions (Fig. 4A). Succinate did not modify basal sTREM2 secretion or the reduction evoked with LPS/IFN‐γ stimulation across any of the lines (Fig. 4A).

Inhibition of glycolysis by 2‐DG led to a 4‐fold increase in basal sTREM2 shedding in Cv iPS‐Mg and a small but not significant increase in TREM2^R47H^ (Fig. 4B). LPS/IFN‐γ evoked shedding in the presence of 2‐DG was still reduced but this was reversed to basal levels by the presence of citrate or succinate (Fig. 4B).

### Stress responses in TREM2 variants are reduced at basal in Cv by citrate or succinate but not modified in R47H or TREM2 ^−/−^

**Fig. 5:**
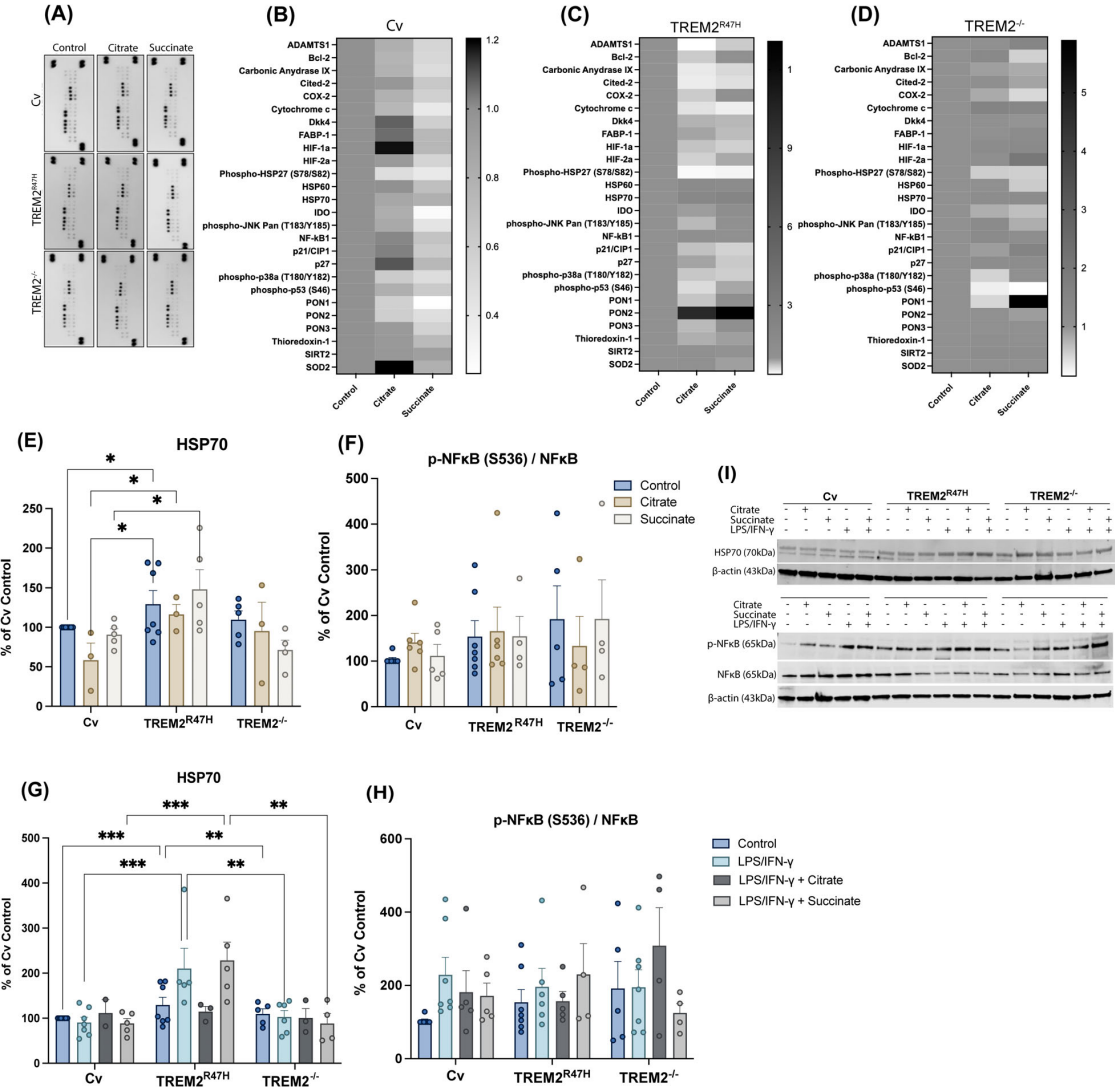
Cellular stress proteome of iPS‐Mg is influenced by citrate or succinate. (A) Representative cellular stress proteome array dot blots and heatmaps from (B) Common variant (Cv), (C) TREM2^R47H^, (D) TREM2^−/−^ variant iPS‐Mg lysates after 24 h incubations with citrate (10 mm) or succinate (10 mm) (*n* = 3–4 biological replicates pooled according to TREM2 genotype after basal or citrate or succinate treatment). (I) Representative western blots and quantifications for HSP70 (E) at basal and (G) upon LPS/IFN‐γ stimulation (*n* = 3–7 biological replicates), and ratio p‐NFκB/NFκB (F) at basal and (H) following LPS/IFN‐γ treatment (*n* = 3–7 biological replicates), in Cv, TREM2^R47H^ and TREM2^−/−^ variant iPS‐Mg after treatments with citrate or succinate. Citrate supplemented Cv iPS‐Mg group is not included in the selected representative blots (I). Data are presented as mean ± SEM. Statistical significance for (E), (F), (G), (H) was addressed using two‐way ANOVA with Tukey *post‐hoc* analysis; *P < 0.05, **P < 0.01, ***P < 0.001.

To investigate the effects of citrate or succinate on cell stress pathways TREM2 LoF iPS‐Mg we used cellular stress proteome arrays (Fig. 5A–D). The total protein expression change in stress proteins in Cv iPS‐Mg was reduced by both citrate and succinate and followed the same trend in TREM2 ^R47H^ but was ineffective in TREM2^−/−^ lines (Fig. 5A–D). To investigate this further, we selected two proteins that showed high expression in all the iPS‐Mg, namely 70 kDa heat‐shock protein (HSP70) and Nuclear factor kappa B (NFκΒ), both of which are implicated in AD and metabolic pathways [31, 32]. Expression levels of HSP70 were not significantly elevated in Cv iPS‐Mg in response to citrate or succinate alone (Fig. 5E,I) or in concert with LPS/IFN‐γ 24 h‐stimulation (Fig. 5G,I). However, the level of HSP70 was significantly elevated at basal in TREM2^R47H^ iPS‐Mg compared with TREM2^Cv^ iPS‐Mg but not modified by citrate or succinate or enhanced significantly by LPS/IFN‐γ (Fig. 5G,I). The effect of citrate or succinate on NFκB activation was measured as the ratio of p‐NFkB to the total NFkB levels on iPS microglia lysates in the absence (Fig. 5E,I) or presence (Fig. 5H,I) of LPS/IFN‐γ treatment. No significant differences were observed upon treatments or among the lines.

### Citrate and succinate effect on synaptosome phagocytosis by iPS‐Mg

**Fig. 6:**
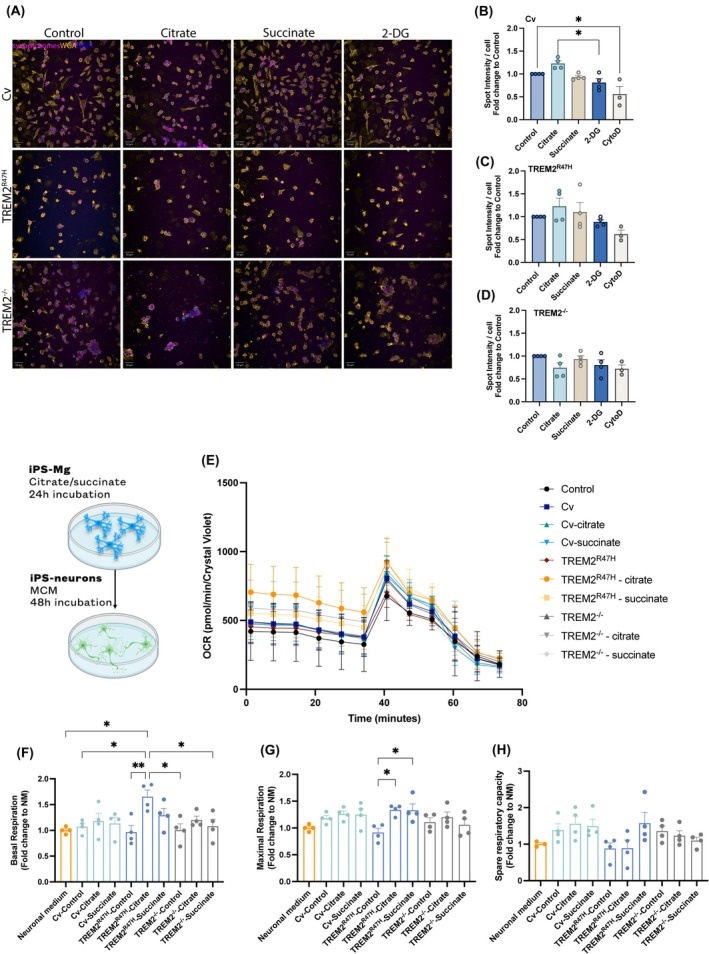
Citrate and succinate effect on synaptosomal uptake by iPS‐Mg and microglia‐regulated changes in neuronal metabolism. (A) Representative images and (B–D) quantification of pHrodo‐labeled synaptosomal uptake by common variant (Cv), TREM2^R47H^ and TREM2^−/−^ iPS‐Mg after treatments with citrate or succinate or 2‐DG glycolytic inhibitor (*n* = 3–4 biological replicates). Scale bars = 100 μm. (E) Metabolic analysis of oxygen consumption rate (OCR), (F) basal respiration, (G) maximal respiration, (H) spare respiratory capacity in control iPS‐neurons and iPS‐neurons incubated for 48 h with MCM media from untreated Cv TREM2^R47H^ and TREM2^−/−^ iPS‐Mg or MCM from citrate or succinate supplemented Cv TREM2^R47H^ and TREM2^−/−^ iPS‐Mg (*n* = 4 iPS‐Mg MCM, *n* = 2 neuronal inductions). Data are presented as mean ± SEM. Statistical significance was addressed for (B), (C), (D), (F), (G), (H) using one‐way ANOVA with Tukey *post‐hoc* analysis; *P < 0.05, **P < 0.01.

Synaptosomal phagocytosis by microglia has been shown previously to be altered in TREM2 variant microglia [33]. Here, we investigated whether medium supplementation with citrate, succinate, or 2‐DG treatment affected synaptosomal phagocytosis in Cv, TREM2^R47H^, and TREM2^−/−^ iPS‐Mg. We did not detect any differences in synaptosome uptake among Cv, TREM2^R47H^, or TREM2^−/−^ lines, although an increasing uptake trend was observed in TREM2^−/−^ iPS‐Mg (Fig. S1D). In Cv iPS‐Mg, synaptosome phagocytosis was not affected by citrate or succinate; however, it was significantly increased in citrate‐supplemented microglia when compared to microglia cells treated with 2‐DG, which blocks glycolysis (Fig. 6A,B). In TREM2^R47H^ or TREM2^−/−^ iPS‐Mg citrate or succinate mediated no significant effects on the uptake of synaptosomes (Fig. 6A,C,D).

### Citrate and succinate‐supplemented microglial conditioned medium rescues TREM2 variant‐induced metabolic deficits in neurons

To test if TREM2 variant microglia affected neuronal bioenergetics and whether citrate or succinate microglia supplementation influenced this process, we incubated iPS‐neurons with MCM from Cv, TREM2^R47H^, or TREM2^−/−^ microglia at basal conditions or previously incubated with citrate or succinate (Fig. 6E–H). First, we detected a decrease in neuronal maximal respiration and spare respiratory capacity mediated by TREM2^R47H^‐Control MCM whereas Cv or TREM2^−/−^‐Control MCM had no significant effect on neuronal oxidative phosphorylation (Fig. S1B). Interestingly, when neurons were incubated with TREM2^R47H^‐Citrate MCM showed increased basal respiration (Fig. 6F) while deficits in maximal respiration induced by TREM2^R47H^‐Control MCM were rescued by both TREM2^R47H^‐Citrate and TREM2^R47H^‐Succinate MCM (Fig. 6G). TREM2^R47H^‐succinate MCM but not TREM2^R47H^‐citrate MCM, was shown to additionally increase spare respiratory capacity to the levels of untreated neurons, although it did not reach statistical significance (Fig. 6H). In iPS‐neurons incubated with MCM from Cv, or TREM2^−/−^ groups we observed no differences in the parameters studied. To rule out the possibility that the observed effects were directly mediated by citrate or succinate which was present in the MCM, we tested the effect of citrate (10 mm) and succinate (10 mm) dissolved in neuronal medium:microglia medium (1 : 1) on neurons. In this setting, neither citrate nor succinate boosted OXPHOS in neurons, which if anything, was compromised by citrate (Fig. S1C).

## Discussion

Metabolic sensing in microglia and other immune cells is an important control of their ability to respond to their surroundings. Deficits in lipid sensing and metabolism are associated with TREM2 LoF variants in microglia [11, 12, 24, 34, 35], and TREM2 deficiency contributes to exacerbating AD pathology *in vivo* by impairing autophagic and metabolic processes [6]. At the same time, TREM2 deficiency has been associated with decreased glycolytic and TCA metabolites *in vitro* [6, 12] suggesting that metabolic and functional deficits displayed by TREM2 LoF variant microglia are attributable to deficits in TCA‐intermediate signaling.

Citrate and succinate are key signaling TCA intermediates and cell cycle breaking points upon insult [19, 36, 37]. Their levels dynamically change during microglia metabolic reprogramming in a disease context‐dependent manner, affecting microglia metabolic status and functions [20, 23, 38]. Here, we investigated whether fueling TREM2 LoF variant iPS‐Mg with citrate or succinate could restore metabolic deficits. We show that citrate is not as effective as succinate at enhancing impaired maximal respiration and spare respiratory capacity in TREM2^R47H^ and TREM2^−/−^ iPS‐Mg, while neither of these substrates significantly modified basal respiration of Cv iPS‐Mg or affected or promoted a glycolytic switch in any of the lines. Previous studies have demonstrated the ability of succinate to boost oxidative phosphorylation in diverse models of diseases characterized by energy dysfunction [39, 40]. Indeed, succinate is an important OXPHOS component that acts synergistically with NADP for electron supply of different complexes of the respiratory chains [41]; thus, less efficient OXPHOS in TREM2^R47H^ and TREM2^−/−^ iPS‐Mg may be linked to either impaired succinate signaling or substrate deficiency in microglia, as it has been reported for macrophages [6]. While both citrate and succinate have been involved in ROS generation or elimination and glial mitochondria function [23, 29, 30, 42] none of the metabolites appeared to alter mitoSOX levels which were higher in TREM2^R47H^ iPS‐Mg reflecting mitochondria abnormalities consistent with our previous findings [12]. Notably, disodium succinate, which we also used in this study as an activator of the extracellular pathway, was reported to mediate no effect on ROS levels or mitochondrial fission in primary microglia in contrast to the cell‐permeable diethyl succinate [16]. Thus, we cannot rule out potential succinate receptor (SUCNR1)‐independent effects on TREM2 LoF‐related mitochondria abnormalities. In addition, while here we selected a concentration of 10 mm for metabolite supplementation [39, 43], it is possible that different concentrations of metabolites could potentially impact microglia metabolic performance, and we cannot entirely rule out the possibility of dose‐dependent effects at other concentrations. Importantly, alterations observed here in proteins involved in mitochondrial stress pathways such as superoxide dismutase 2 (SOD2), or hypoxia‐inducible factor 1 α (HIF1α) upon succinate or citrate supplementation that we detected across the lines in the proteome array, are suggestive of an impact on mitochondria function possibly attributable to their impact on OXPHOS that is perturbed in TREM2 LoF variants. Meanwhile, studies have pointed out that it is the immunometabolic shifts that draw a link between TREM2 dysregulation and a disease‐associated microglia (DAM) profile [44]. We show that citrate and succinate supplementation of microglia led to an overall decrease of the cell stress proteome, suggesting that TCA metabolites may be beneficial for microglia and compensate for energy deficits due to TREM2 loss of function. Indeed, succinate supplementation was shown to be protective in a mixed microglia culture model of mitochondria dysfunction and in TBI patients [39, 40].

TREM2 is a modulator of lipid metabolism, and the R47H variant of TREM2 has reduced affinity for apolipoproteins (e.g., apolipoprotein E; APOE) or lipid ligands, which may contribute to the increased late‐onset AD risk associated with this TREM2 variant [24, 35]. This has been suggested as a consequence of the inability of the microglia to process lipids sufficiently, leading to chronic lipid accumulation, particularly of myelin and APOE [5, 34, 45, 46, 47, 48]. Our study shows that TREM2^R47H^ and TREM2^−/−^ iPS‐Mg displayed an overall lower lipid accumulation under basal conditions, but when treated with citrate, and to a lesser extent with succinate, the lipid levels increased to those of Cv. These findings suggest that driving OXPHOS in these cells promotes lipid accumulation to levels observed in Cv cells. Furthermore, it is substantiated that increased citrate availability leads to fatty acid synthesis and lipogenesis, which, although detrimental in healthy conditions, is disturbed in neurodegenerative diseases [30]. This is further corroborated by the observed effect of citrate and succinate on metabolically healthy iPS‐Mg, mediating either no change (citrate) or decreased (succinate) lipids levels in contrast with TREM2 LoF iPS‐Mg. Although it has been proposed that chronic lipid accumulation occurs in models of TREM2^−/−^ [35, 49], recent evidence suggests a downregulation of lipid metabolism in microglia expressing the TREM2 LoF pQ33X mutation associated with Nasu‐Hakola disease [50] in accordance with our results. We previously found that exposure of TREM2^R47H^ variant microglia to cells expressing phosphatidylserine (PS+), ameliorated some of the mitochondrial deficits we reported [12, 13], and we suggested that where lipid signaling is used to compensate for lack of energy, the R47H variant may prevent ‘efficient’ use of a lipid energy source.

In line with the lack of apparent effects on glycolysis delivered by citrate or succinate and due to their ability to primarily influence OXPHOS, supplementation did not affect the production of the glycolytic by‐product, lactate, in our model. However, extracellular lactate levels were markedly increased upon LPS/IFN‐γ induced microglia activation, consistent with previous *in vitro* findings [51, 52]. During glycolysis, pyruvate is turned into the TCA by‐product lactate anaerobically, stimulated by PI3K/Akt signaling. However, no differences in the detected lactate levels upon supplementation cannot be interpreted as a lack of effect on this glycolytic process. Previous studies show that succinate supplementation altered the lactate/pyruvate ratio, a clinical marker of tissue cerebral metabolic state in disease, *in vivo* and *in vitro*, without significant differences in lactate concentration following succinate treatments [39, 40]. It is also important to note that lactate release was higher in TREM2^R47H^ and TREM2^−/−^ lines when compared with Cv iPS‐Mg in basal conditions and upon LPS/IFN‐γ stimulation. It is, therefore, likely that the reported glycolytic deficits of TREM2 deficient microglia are associated with disruptions during glucose conversion into ATP through pyruvate production that result in lactate accumulation. Recently, increased lactate levels were reported in the microglia of AD mouse models characterized by amyloidosis, and a glycolysis/H412 lactylation/PKM2 positive feedback loop in microglia that exacerbates glucose metabolism disorder and proinflammatory activation of microglia in AD [53]. These results point to a role of TREM2 signaling in microglial lactate metabolism which deserves further exploration.

To understand further the functional consequences of energy substrate supplementation, we analyzed the ability of the cells to phagocytose pathogenic insults such as Aβ_1–42_ and secrete cytokines, functions which we have previously shown to be impaired in R47H variant iPS‐Mg [12]. Here, citrate and succinate supplementation restored the deficient phagocytic capacity in TREM2^R47H^ (citrate) or TREM2^−/−^ (citrate, succinate), suggesting that boosting OXPHOS (succinate) and/or lipid metabolism (citrate, succinate) was able to compensate for energy deficits in the cells. Upon inhibition of glycolysis, we showed reduced Aβ_1–42_ phagocytosis by Cv iPS‐Mg. Conversely, the reduced phagocytic capacity of TREM2^R47H^ and TREM2^−/−^ iPS‐Mg was unaffected by 2‐DG inhibition, demonstrating Cv iPS‐Mg primary dependency on glycolysis to respond to pathology which is not the case for the TREM2 LoF variant microglia. In this setting, citrate reversed 2‐DG‐induced phagocytic impairments in Cv iPS‐Mg, further supporting the hypothesis that substrate supplementation can supply the cells with the necessary energy upon metabolic stress. The effect of the substrates on the pro‐inflammatory response was less evident even though citrate and succinate have been reported to be pro‐inflammatory agents *per se*, along with existing evidence suggesting a pro‐inflammatory response upon activation of the succinate receptor (SUCNR1) in the presence of extracellular succinate [16, 29, 30]. While citrate or succinate supplementation did not affect iPS‐Mg basal cytokine secretion to detectable levels, both substrates promoted a subtle pro‐inflammatory microglia response following LPS/IFN‐γ stimulation. Succinate has been shown to lead to sustained IL‐1β production through HIF1‐α stabilization [29]. Our data point to larger effects of succinate and citrate on cytokine secretion by TREM2^R47H^ and TREM2^−/−^ cells than Cv, and are in line with the observed effects of the substrates on OXPHOS. Upon inhibition of glycolysis by 2‐DG and LPS/IFN‐γ‐evoked TNFα, IL‐1β secretion did not decrease as previous studies have reported [54, 55], but instead, their secretion was further amplified in the presence of substrates, suggesting that alternative pathways other than glycolysis may also drive pro‐inflammatory cytokine secretion in the cells. There is a significant interplay between cytokines and glucose/lipid metabolism [56], regarding the influence of cytokines on these processes. Altered glucose metabolism can affect cytokine production from human peripheral blood mononuclear cells (PBMCs) and the human monocyte cell line, THP‐1 [57].

TREM2 undergoes ectodomain shedding by ADAM10/17 proteases, resulting in the release of soluble sTREM2. In AD, sTREM2 has been suggested to either indicate reduced TREM2 signaling and thus impaired microglia functions, or to be protective [58]. The relationship between sTREM2 release and metabolic fitness is poorly explored. First, we found that LPS/IFN‐γ resulted in decreased sTREM2 shedding in culture supernatant, suggesting that TREM2 shedding is decreased upon LPS/IFN‐γ‐induced glycolytic switch. However, given that pro‐inflammatory conditions, such as LPS/IFN‐γ stimulation, have been previously shown to reduce TREM2 expression—resulting in decreased sTREM2 release and detection [59]—we further evaluated sTREM2 shedding after blocking glycolysis with 2‐DG and found that its levels correspondingly increased. These results show for the first time that immunometabolic processes may influence TREM2 processing, while this did not seem to be affected by citrate or succinate supplementation in basal conditions. Upon concomitant 2‐DG and LPS/IFN‐γ stimulation, sTREM2 levels tended to be increased by citrate and to a lesser extent by succinate suggesting that alternative sources of energy provided by substrate fueling of the cells upon stress insults influence the sTREM2 shedding process in Cv iPS‐Mg. Similar trends were observed in different conditions in TREM^R47H^ iPS‐Mg, which further support this hypothesis even though they did not reach significance, likely due to the already low basal sTREM2 levels in TREM2^R47H^ lines. Considering that recent data highlight the role of sTREM2 not only in microglial functions but also in microglia‐neuronal interactions in AD [60, 61] understanding the link between sTREM2 and immunometabolism could provide new insights into the pathogenesis of AD to support the development of new therapies.

Tagliatti *et al*., [62] recently explored the potential role of TREM2 in shaping neuronal bioenergetics, showing that lack of TREM2 leads to changes in the metabolomic profile on the mouse hippocampus during development. In line with this evidence, we have previously shown that exosomes secreted by TREM2^R47H^ variant iPS‐Mg exhibit an altered proteome, particularly related to metabolic pathways, and are less effective in supporting neuronal viability against toxic insults compared to exosomes secreted by common variant iPS‐Mg [14, 15]. Here, we show for the first time that the secretome of TREM2^R47H^ iPS‐Mg directly impairs neuronal OXPHOS and in turn, citrate and succinate supplementation of TREM2^R47H^ iPS‐Mg mitigates these deficits. This evidence suggests that metabolic manipulations, such as nutrient supplementation, that can restore microglia metabolic and functional deficiencies due to the R47H variant, may harness their ability to support neuronal metabolism, and thus prevent neurodegeneration, but the exact mechanisms need to be further investigated. Our results for TREM2^−/−^ are somewhat confusing in this context, but since this is an extreme phenotype in view of the loss of all TREM2 expression, the cells may in some way be able to compensate, and this was reflected across a number of our assays. Besides neuronal function, TREM2 has been involved in synaptic formation and elimination through microglia phagocytosis in a context‐dependent manner [33, 63, 64]. While these effects are thought to rely—at least in part‐ on TREM2 ability to regulate microglial OXPHOS [65], in our setting, and contrast to the observed effects on Aβ phagocytosis, neither citrate nor succinate altered synaptosomal phagocytosis by microglia. These results highlight the complexity of the dynamics of phagolysosomal uptake and digestion of Aβ versus synaptic material and further research is needed to elucidate the precise mechanisms underlying these observations.

**Fig. 7:**
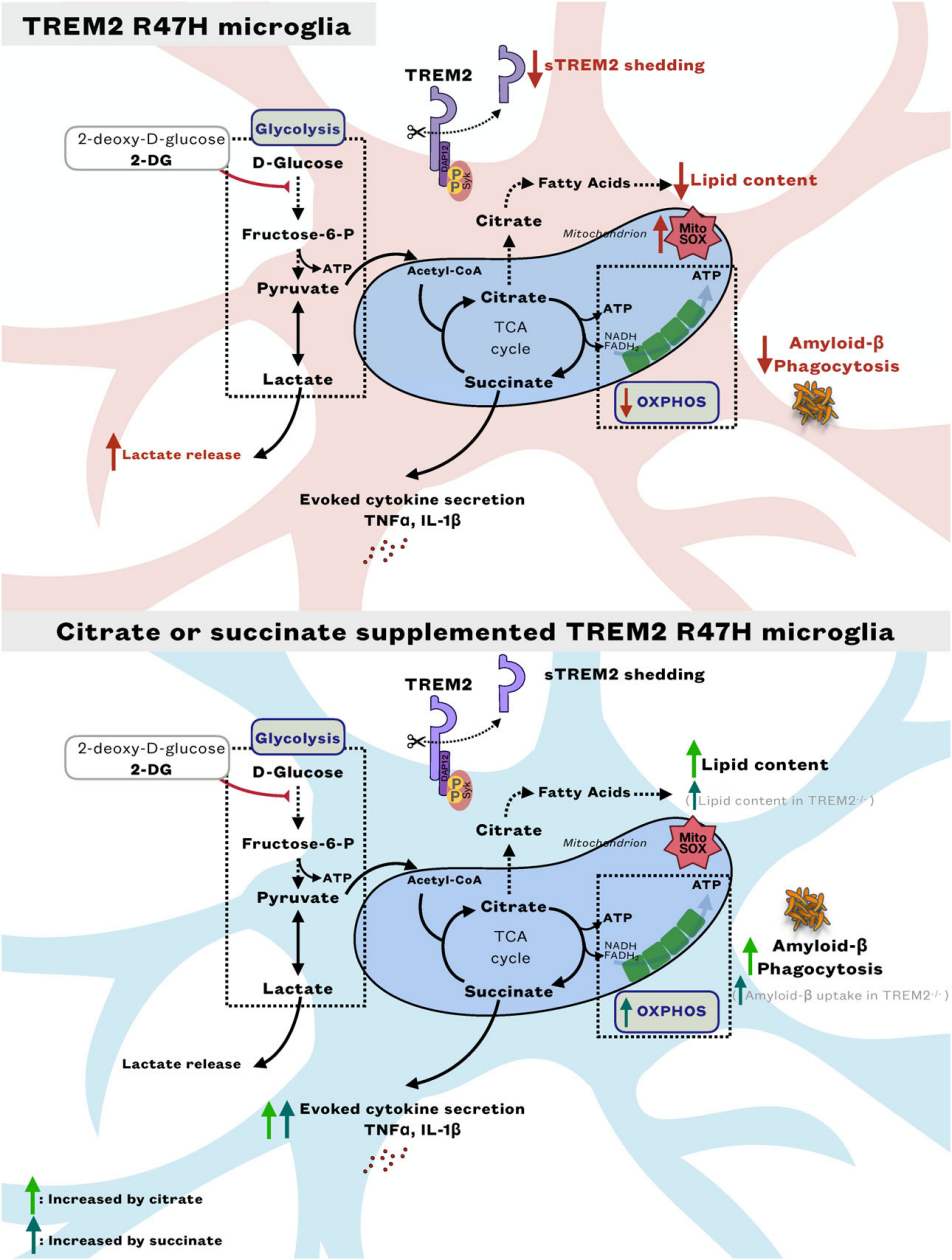
Amelioration of signaling deficits in TREM2^R47H^ iPS‐Mg after supplementation with citrate or succinate. Microglia metabolic shortfall and functional deficits, including deficits in oxidative phosphorylation (OXPHOS), lipid metabolism and amyloid‐β phagocytosis due to TREM2^R47H^ are ameliorated when fuelling TREM2 deficient iPS‐microglia with the TCA cycle metabolites citrate or succinate. Particularly, citrate increases lipid content and amyloid‐β uptake in TREM2^R47H^ microglia, while succinate restores deficits in OXPHOS exhibited by TREM2^R47H^ microglia and increases amyloid‐β uptake in TREM2^−/−^ microglia. Both metabolites promote a slight increase in inflammatory cytokine secretion in LPS/IFNγ‐activated human TREM2^R47H^ iPS‐microglia. Αmelioration of TREM2^R47H^ microglia shortfall by citrate or succinate impacts on neuronal metabolism via secretome related pathways.

Our results show that TCA metabolite signaling pathways are perturbed in TREM2 LoF variant iPS‐Mg contributing to the observed metabolic and functional deficits displayed by those cells (Fig. 7), providing insights into the relationship between dysfunctional metabolism and TREM2 with implications for AD pathogenesis and treatments.

## Materials and methods

### Human iPSC generation of microglia and neurons

Ethical permission for this study was obtained from the National Hospital for Neurology and Neurosurgery and the Institute of Neurology joint research ethics committee (study reference9/H0716/64). The TREM2^R47H^ homozygous line (BIONi010‐C7; RRID:CVCL_II86) and TREM2^−/−^ line (BIONi010‐C17; RRID:CVCL_RM88) were gene‐edited BioNi010‐C (RRID:CVCL_1E68), purchased from EBiSC. SFC840 (Stembancc; STBCi026‐B; RRID:CVCL_RB86) and BioNi010‐C were used as the common variant (Cv) control lines. Human induced pluripotent stem cells (iPSC) were maintained and routinely passaged in Essential 8 medium. All cell lines were mycoplasma‐tested before use according to the manufacturer's instructions (Mycoalert Detection Kit, Lonza, Basel, Switzerland), and all cell lines were authenticated by Sanger sequencing for variant expression within the last 3 years.

Using our previously described protocol [12], which incorporates procedures from earlier protocols [11, 66] iPSC‐derived microglia were generated. Experimental replicates were one clone assayed in at least three independent experimental runs (Cv Control, TREM2^R47H^ homozygous, and TREM2^−/−^). For characterization of the differentiated iPSC‐microglia, we carried out immunohistochemistry on cells with known microglial markers, as a quality control, based on our previous papers. The cells were plated and matured on 13 mm glass coverslips. The cells were fixed with 4% PFA in PBS, unspecific binding sites were blocked with 5% normal goat serum, and the cells were incubated with primary antibodies for staining of established microglia markers, including IBA‐1 (019‐19741, Wako, Merck, Darmstadt, Germany), P2RY12 (HPA014518, Atlas Antibodies, Stockholm, Sweden), TMEM119 (HPA051870, Atlas Antibodies). The cells were then incubated with anti‐rabbit AlexaFluor 488 (Invitrogen, Waltham, MA, USA) conjugated secondary antibodies and nuclei were stained with DAPI. Different regions per coverslip were imaged on a Zeiss LSM710 confocal microscope (63×) using the lsm pascal 5.0 software, Zeiss, Oberkochen, Baden‐Württemberg, Germany (Fig. S3).

Differentiation of the iPSC (BioNi010‐C line) into cortical neurons was performed following a published protocol [67] with slight modifications. In brief, confluent iPSC was switched to a neuronal maintenance media (NMM), supplemented with 10 μm SB431542 (Tocris, Bristol, UK) and 200 nm LDN‐193189. (Tocris). NMM consisted of 1 : 1 ratio of Dulbecco's modified eagle medium F12 (DMEM‐F12) and Neurobasal supplemented with 0.5× B27, 0.5× N2, 1.7× GlutaMAX, 0.5× sodium pyruvate, 25 μg·mL^−1^ Pen/Strep, 50 μm 2‐mercaptoethanol, 0.5× nonessential amino acids, 2.5 μg·mL^−1^ insulin. On day 12, neuroepithelial cells were passaged to laminin‐coated plates, and the medium was switched to NMM. On day 33 cells were plated in laminin/poly‐l‐ornithine (PLO) coated plates. For characterization of the differentiated iPSC‐neurons, we carried out immunohistochemistry on these cells with known neuronal markers, as a quality control. iPS‐neurons were seeded and matured on 13 mm glass coverslips. On day 100, the cells were fixed with 4% PFA in PBS, unspecific binding sites were blocked with 5% normal goat serum and the cells were incubated with primary antibodies for staining of established neuronal markers, including TUJ1 (Biolegend, San Diego, CA, USA), TBR1 (EPR8138, Abcam), BRN2 (B‐2, Santa Cruz Biotechnology, Dallas, TX, USA), SATB2 (SATBA4B10, Abcam), and Synaptophysin (YE269, Abcam). The cells were then incubated with anti‐mouse or anti‐rabbit AlexaFluor 488 or 568 (Invitrogen) conjugated secondary antibodies and nuclei were stained with DAPI. Different regions per coverslip were imaged on a Zeiss LSM710 confocal microscope (40×, 63×) using the lsm pascal 5.0 software. Brightfield images were obtained at critical points of the neuronal differentiation using a Leica microscope (20×) (Fig. S1A).

### Cellular energy analysis

For real‐time analysis of oxygen consumption rates (OCR) (using Seahorse XF Cell Mito Stress Test kits), or proton efflux rate (PER; using Seahorse Glycolytic Rate assays, all Agilent Technologies), iPS‐Mg were plated (20 000–25 000 per well), matured on Seahorse cell culture microplates and analyzed using a Seahorse XFe96 Analyzer (Agilent Technologies, Stockport, Cheshire, UK) as previously described [12, 13]. Where indicated, cells were incubated with 10 mm succinate (succinic acid, disodium salt, Sigma, Merck, Darmstadt, Germany) or 10 mm citrate (sodium citrate, trisodium salt, Sigma) in the presence or absence of 100 ng·mL^−1^ Lipopolysaccharide (LPS) and 10 U·mL^−1^ interferon‐gamma (IFN‐γ) for 24 h before analysis. Data were analyzed using wave v2.4.0.6 software (Agilent Technologies) upon Crystal Violet normalization.

For real‐time analysis of OCR in neurons, iPS‐neurons were plated (50 000 cm^−2^) on laminin/PLO‐coated Seahorse cell culture microplates and matured until day 100. On day 98, iPS‐neurons were incubated for 48 h with microglia‐conditioned medium (MCM; 1 : 1 ratio of microglia medium to neuronal medium) from untreated Cv, TREM2^R47H^, or TREM2^−/−^ microglia and MCM after 24 h incubation of Cv, TREM2^R47H^ or TREM2^−/−^ microglia with citrate (10 mm) or succinate (10 mm). Data were analyzed using wave v2.4.0.6 software (Agilent Technologies) upon Crystal Violet normalization.

### ELISA analysis of cytokine secretion and shed soluble TREM2

IPS‐Mg (50 000 cm^−2^) were stimulated with LPS/IFN‐γ (100 μg·mL^−1^ and 10 U·mL^−1^, respectively) for 24 h in the presence of citrate (10 mm), 2‐deoxyglucose (2‐DG, 3 mm), citrate plus 2‐DG, or succinate (10 mm) alone or with 2‐DG. Cell culture medium was collected, centrifuged at 300 ***g*** for 15 min to remove cell debris, and typically 50 μL (1 : 2 dilution) appraised for secreted TNFα or 200 μL (1 : 2 dilution) for secreted IL‐1β using human Quantikine ELISA kits, as per the manufacturer's instructions (R&D, Abingdon, UK).

Soluble TREM2 (sTREM2) was appraised in 100 μL of cell culture medium with an in‐house‐generated ELISA system as previously described [11, 13]. Briefly, MaxiSORP 96‐well plates were coated with 1 μg·mL^−1^ of a rat anti‐mouse/human TREM2 monoclonal antibody (R&D Systems; clone 237920) at 4 °C, overnight. After blocking supernatant samples from different groups and standards (recombinant human‐TREM2‐His; Life Technologies, Invitrogen, Thermo Fisher Scientific, Waltham, MA, USA) were incubated for 2 h at room temperature (RT) with biotinylated polyclonal goat anti‐human TREM2 capture antibody (0.1 μg·mL^−1^; AF1828, R&D Systems). Following incubation with streptavidin‐HRP (Invitrogen; 0.1 μg·mL^−1^), chromogenic substrate solution was added (TMB, Life Technologies) the reaction terminated (with 0.16 m H_2_SO_4_) and absorbance read at 450 nm (EZ Read microplate). Values were normalized to the protein content of cell lysates for each sample.

### Lipid spot staining

IPS‐Mg (60 000/coverslip) were matured on 13 mm glass coverslips. Cells were treated with citrate (10 mm) or succinate (10 mm) for 24 h and subsequently fixed with 4% PFA in PBS for 20 min at RT. For lipid spot staining, cells were washed with PBS, incubated with Wheat germ agglutinin‐594 (WGA, ThermoFisher Scientific, Waltham, MA, USA) (1 : 2000) for 5 min, following permeabilization with 0.1% Triton‐X. Cells were washed with PBS and subsequently incubated with LipidSpot™488 (Biotium; Cambridge BioScience, Cambridge, UK) (1 : 1000) for 15 min in the dark. Nuclei were stained with DAPI and coverslips were mounted on glass slides using Fluoromount‐G (Invitrogen). Three to five different regions per coverslip were imaged using a Zeiss LSM710 confocal microscope using the lsm pascal 5.0 software.

### Lactate release in cell culture medium

Levels of lactate secreted from iPS‐Mg into the supernatant were determined using an l‐lactate Assay kit (Abcam) and a d‐lactate assay kit (Abcam), after 24 h treatment with citrate (10 mm) or succinate (10 mm), or 2‐DG, (3 mm) in the presence or absence of LPS/IFN‐γ (100 ng·mL^−1^ and 10 U·mL^−1^, respectively) as per the manufacturer's instructions. Values were normalized to the protein content of cell lysates for each sample.

### Mitochondrial superoxide analysis

Mitochondrial superoxide levels were measured by loading iPS‐Mg (50 000 cm^−2^) with MitoSOX™ red superoxide indicator (ThermoFisher) and analyzed by flow cytometry as previously described [12]. Following 24 h treatments with citrate (10 mm) or succinate (10 mm), cells were incubated with MitoSOX™ red at 5 μm in PBS + 0.5% BSA for 10 min at 37 °C. 200 000 to 300 000 cells per treatment group were washed and resuspended in PBS followed by flow cytometric analysis (FL2; FACS Calibur, Becton Dickinson, Wokingham, Berkshire, UK). As a positive control for mitochondrial superoxide generation, cells were incubated with rotenone (100 nm) for 30 min before the addition of MitoSOX. Data were analyzed using flowing Software v2.5.2 (University of Turku).

### Western blotting

iPS‐Mg (50 000 cm^−2^) were lysed in RIPA buffer (50 mm Tris, 150 mm NaCl, 1% SDS, and 1% Triton X‐100) containing 1× Halt™ protease and phosphatase inhibitor cocktail at 4 °C. Lysates were centrifuged at 15 000 ***g*** for 15 min at 4 °C to clarify the lysate and separate the soluble and insoluble (nuclear) fractions. Fractions were snap‐frozen on dry ice and stored at −80 °C until use. Soluble fractions were normalized after BCA protein quantification and 4× running buffer (LiCor, Cambridge, UK) plus 10% DTT. Samples were denatured at 95 °C for 10 min and run on 4–20% Criterion TGX Precast Midi Protein gels (Bio‐Rad, Watford, Hertfordshire, UK). Resolved protein was transferred to nitrocellulose using Trans‐Blot turbo transfer packs, washed for 10 min at RT with TBS, and blocked for 1 h at RT in 5% milk in TBS‐T. Blocked membranes were incubated in primary antibody overnight at 4 °C (Phospho‐NF‐κB p65 (Ser536) (93H1), Cell Signaling (Cell Signaling Technology, Danvers, MA, USA), (1 : 1000); NF‐κB p65 (D14E12), Cell Signaling, (1 : 1000); Anti‐β‐Actin (AC‐15), Sigma Millipore (1 : 5000); Hsp70 (5A5), Abcam, (1 : 500)). Membranes were washed with TBS‐T and incubated for 1 h at RT in the dark with corresponding LiCor compatible Alexafluor 680/800 nm conjugated secondary antibodies or HRP‐conjugated anti‐mouse or anti‐rabbit (ECL detection). The membranes were washed 3× with TBS‐T followed by a final TBS‐T wash and visualized using an Odyssey detection system (LiCor). Protein bands were subsequently quantified using exported zip files in image studio lite v5.2 (LiCor, Cambridge, UK).

### Cell stress array analysis

IPS‐Mg were incubated for 24 h with citrate (10 mm) or succinate (10 mm) and cell lysates were collected and prepared according to manufacturer's instructions (Proteome Profiles™ Human cell stress array; Bio‐Techne, Bristol, UK). Total protein quantification was performed on the aliquots of each treatment group for data normalization purposes. Cell lysates from 3 to 4 independent experiments were pooled according to the TREM2 genotype after basal or citrate/succinate treatment. Data were analyzed and quantified using exported zip files in image studio lite v5.2. Data were plotted as relative protein expression, normalized to total cellular protein levels.

### Aβ_1–42_ (HiLyte488) phagocytosis

IPS‐Mg were plated at a density of 50 000 cm^−2^ and were pre‐treated with citrate (10 mm), succinate (10 mm), and 2‐DG (3 mm) for 24 h. Oligomerized Aβ_1–42_ (HiLyte488; 100 nm) was added to all groups and cells were incubated at 37 °C + 5% CO_2_ for 2 h. Cytochalasin‐D (CytoD; 100 μm) was added to negative control groups for 30 min before Aβ_1–42_ addition. 200 000 to 300 000 cells were then collected, centrifuged at 300 ***g*** for 3 min at RT, and the cell pellets were resuspended in PBS for FACS analysis (FL1; FACs Calibur, Beckton Dickinson). Data were analyzed using flowing software v2.5.1 (Turku Centre of Biotechnology, University of Turku).

### Synaptosomal phagocytosis

Synaptosomes were a kind gift from Professor Guy Brown, University of Cambridge, and were labeled with pHrodo and incubated with iPS‐Mg as previously described [33]. Briefly, iPS‐Mg (20 000 per well) were plated and matured on 96‐well plates suitable for imaging with the Opera Phenix high‐content screening system. Cells were pre‐incubated with citrate (10 mm), succinate (10 mm) and 2‐DG (3 mm) 24 h before performing the uptake experiments. On the day of the experiment, the synaptosomes were thawed, resuspended in Hepes‐buffered saline solution (HBSS) and incubated with pHrodo (10 μm) for 30 min at 37 °C. After incubation, synaptosomes were washed thrice with buffer and 2 μg of pHrodo‐labeled synaptosomes were added to 20 000 iPS‐Mg for 2 h 37 °C to evaluate uptake by the cells. Subsequently, the cells were washed with HBSS twice and incubated with 5 μg·mL^−1^ WGA‐488 (ThermoFisher) and DAPI for cell membrane and nuclei staining, respectively. Cytochalasin D (Cyto D, 100 μm) as a negative control of phagocytosis, was added for 30 min before the addition of pHrodo‐labeled synaptosomes. Cells were imaged live using Opera Phenix microscopy (20×), and analysis was performed using the columbus software. For analysis, the sum of the corrected mean intensity of the ‘spots’ detected within the cell cytoplasm was divided by the number of cells (four biological replicates, four technical replicates (wells), eight fields per well).

### Data analyses and statistics

All experiments were performed on at least three independent cell plantings with technical replicates of at least three per experiment. Where indicated data were normalized to cell protein content (BCA) or cell number (Crystal violet). For cell proteome arrays, data were analyzed on pooled samples from three different cell platings. Data are presented as mean ± SEM (*n* = 3–12). Statistical significance was addressed using one‐way ANOVA or two‐way ANOVA with Tukey *post‐hoc* analysis to compare controls and treated groups, respectively, unless otherwise stated. Statistical significance was considered when *P*‐values were < 0.05.

## Conflict of interest

The authors declare no conflict of interest.

## Author contributions

JMP, TMP, and FV designed the study, FV and TMP carried out the experiments and analyzed the data, WJ performed additional data analyses, JMP, FV, and TMP wrote the paper. All authors provided comments, contributed to, and agreed on the final version of the paper.

## Supporting information


**Fig. S1.** Characterization of iPS‐neurons, metabolic analysis and synaptosome uptake in control groups.
**Fig. S2.** Additional data in OCR and cytokine secretion upon LPS/IFN‐γ stimulation.
**Fig. S3.** Characterization of iPS‐Mg by immunocytochemistry.

## Data Availability

All data are available on request.
