# Supplementary figures and images for: Amelioration of signaling deficits underlying metabolic shortfall in TREM2R47H human iPSC‐derived microglia
Source: FEBS J. 2024 Dec 26;292(7):1743–62. doi: 10.1111/febs.17353 (PMC11970715; doi:10.1111/febs.17353)

**Supplementary Figure S1**


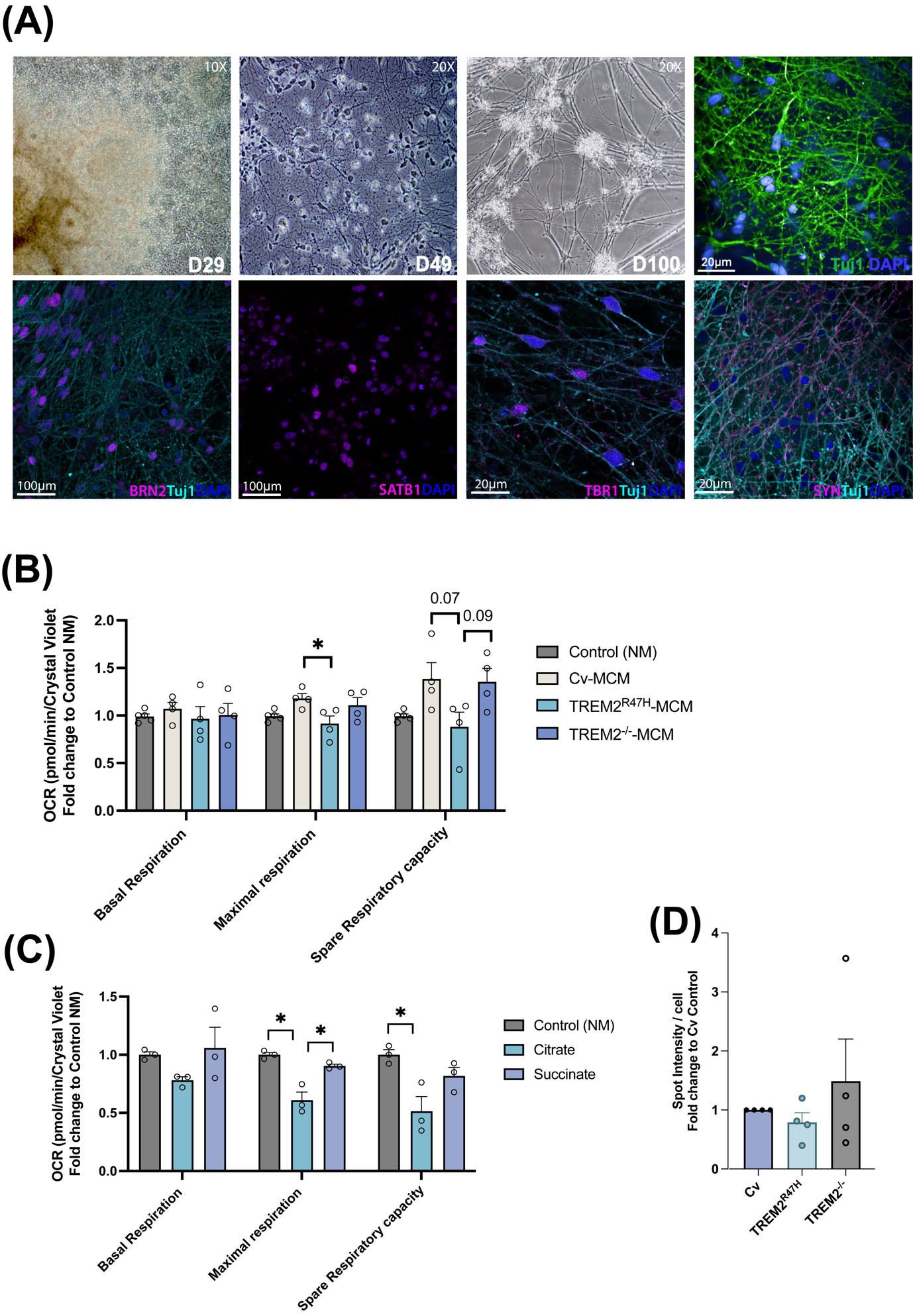


**Supplementary Figure S2**


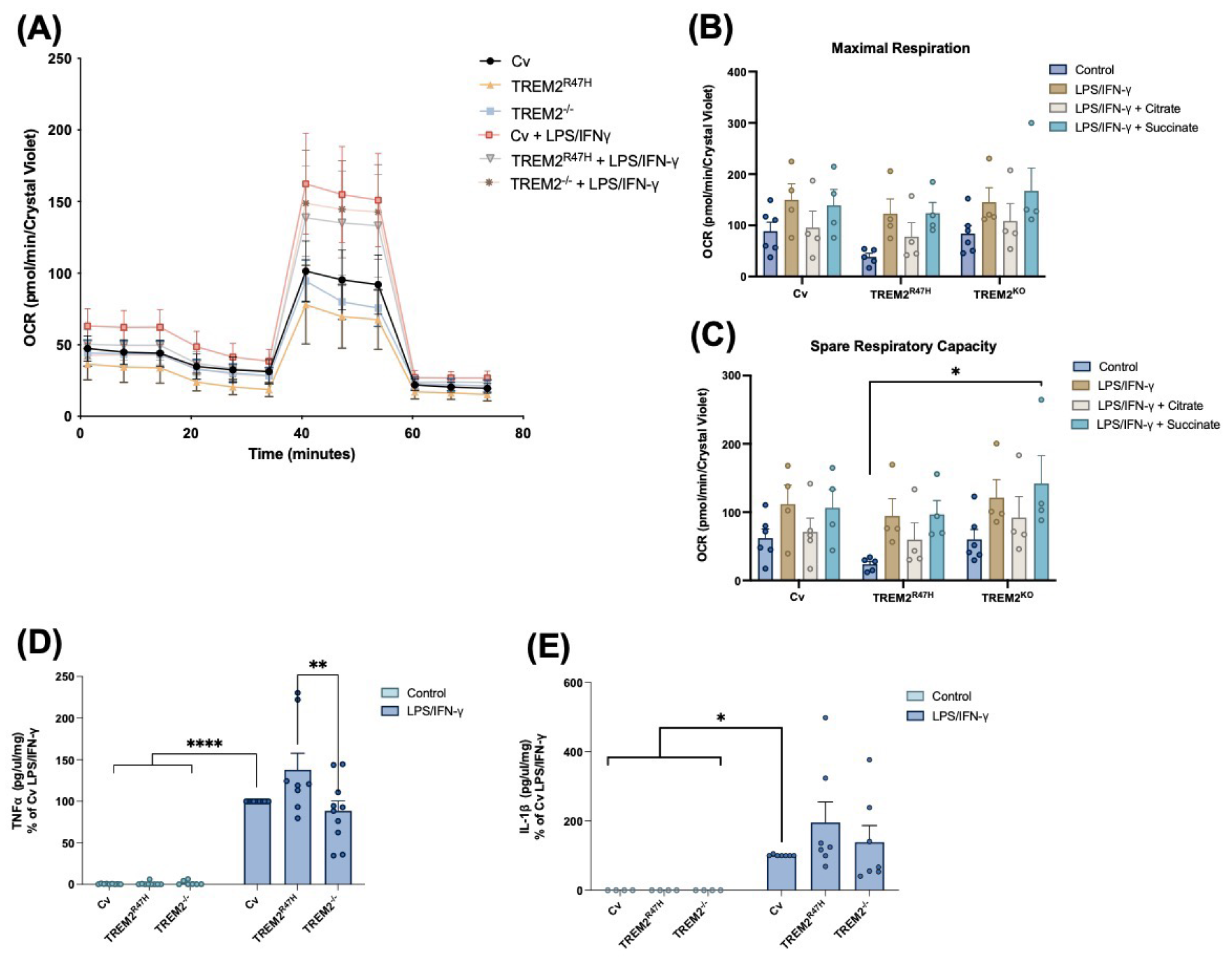


**Supplementary Figure S3**


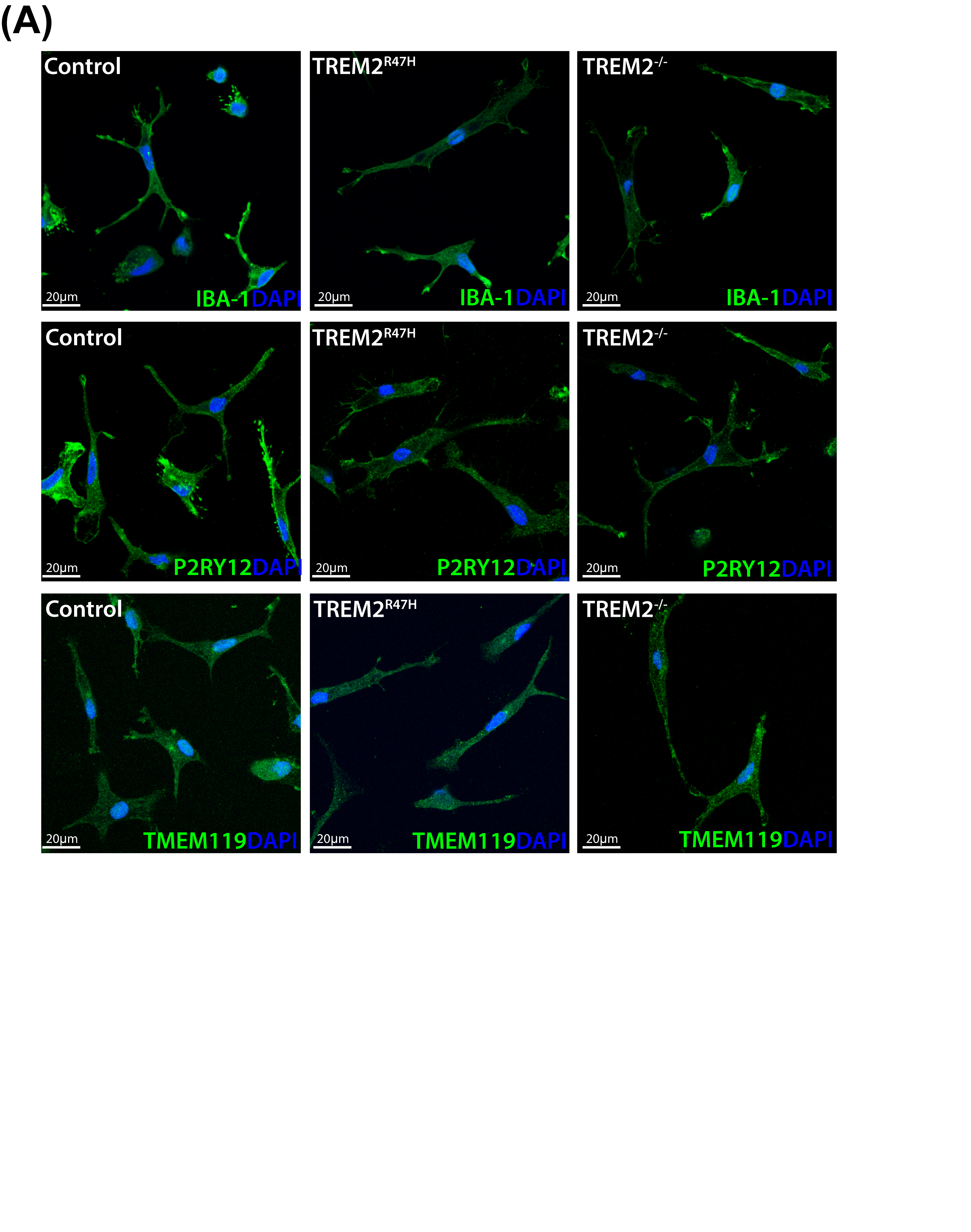

Supplement: Supplementary file 1 — Fig. S1. Characterization of iPS‐neurons, metabolic analysis and synaptosome uptake in control groups. Fig. S2. Additional data in OCR and cytokine secretion upon LPS/IFN‐γ stimulation. Fig. S3. Characterization of iPS‐Mg by immunocytochemistry. [file FEBS-292-1743-s001.docx]
